# Adhesion molecule gene variants and plasma protein levels in patients with suspected obstructive sleep apnea

**DOI:** 10.1371/journal.pone.0210732

**Published:** 2019-01-17

**Authors:** Andrew J. Sandford, Amanda Ha, David A. Ngan, Loubna Akhabir, Aabida Saferali, Nurit Fox, A. J. Hirsch Allen, Simon C. Warby, Stephan F. van Eeden, Najib T. Ayas

**Affiliations:** 1 Centre for Heart Lung Innovation, St. Paul's Hospital and University of British Columbia, Vancouver, British Columbia, Canada; 2 UBC Hospital Sleep Disorders Program, Division of Respiratory Medicine, University of British Columbia, Vancouver, British Columbia, Canada; 3 Center for Advanced Research in Sleep Medicine, Centre de Recherche de l'Hôpital du Sacré-Cœur de Montréal, Montréal, Québec, Canada; 4 Département de Psychiatrie, Université de Montréal, Montréal, Québec, Canada; 5 Division of Critical Care Medicine, University of British Columbia, Vancouver, British Columbia, Canada; 6 Center for Health Evaluation and Outcome Sciences, St. Paul's Hospital and University of British Columbia, Vancouver, British Columbia, Canada; Osaka University Graduate School of Medicine, JAPAN

## Abstract

**Study objectives:**

Untreated obstructive sleep apnea (OSA) patients have an increased risk of cardiovascular disease (CVD). Adhesion molecules, including soluble E-selectin (sE-selectin), intercellular adhesion molecule-1 (ICAM-1), and vascular adhesion molecule-1 (VCAM-1), are associated with incident CVD. We hypothesized that specific genetic variants will be associated with plasma levels of adhesion molecules in suspected OSA patients. We also hypothesized that there may be an interaction between these variants and OSA.

**Methods:**

We measured levels of sE-selectin, sICAM-1 and sVCAM-1 in 491 patients with suspected OSA and genotyped them for 20 polymorphisms.

**Results:**

The most significant association was between the *ABO* rs579459 polymorphism and sE-selectin levels (*P* = 7×10^−21^), with the major allele T associated with higher levels. The direction of effect and proportion of the variance in sE-selectin levels accounted for by rs579459 (16%) was consistent with estimates from non-OSA cohorts. In a multivariate regression analysis, addition of rs579459 improved the model performance in predicting sE-selectin levels. Three polymorphisms were nominally associated with sICAM-1 levels but none with sVCAM-1 levels. The combination of severe OSA and two rs579459 T alleles identified a group of patients with high sE-selectin levels; however, the increase in sE-selectin levels associated with severe OSA was greater in patients without two T alleles (*P* = 0.05 test for interaction).

**Conclusions:**

These genetic polymorphisms may help to identify patients at greatest risk of incident CVD and may help in developing a more precision-based approach to OSA care.

## Introduction

Obstructive sleep apnea (OSA), characterized by recurrent collapse of the upper airway during sleep, is a common under-diagnosed disease that causes serious individual and population health consequences. Untreated OSA can cause disabling symptoms, increase health care utilization, lead to automobile crashes and injuries, and to premature death [1]. In a community-based study of middle-aged subjects, 9% of men and 4% of women had moderate to severe OSA (i.e., apnea hypopnea index (AHI) ≥15/hr) [2]. Based upon the subsequent increased prevalence of obesity, current estimated OSA rates are 14–55% higher [3]. The vast majority of patients have not been clinically diagnosed [4].

Untreated patients with OSA have a three-fold increased risk of incident cardiovascular disease (CVD) including strokes and heart attacks, after controlling for confounders such as age, smoking, and hypertension [5]. One mechanism by which OSA could lead to premature CVD is the activation of systemic inflammation via increased oxidative stress, through hypoxia inducing factor or activation of Nuclear Factor-κB (NFκB) [6, 7]. Inflammation plays a key role in the pathogenesis of atherosclerosis in non-OSA populations [8, 9]. Furthermore, cell adhesion molecules (CAMs), which modulate the binding and recruitment of leukocytes to the vascular endothelium, are present in atherosclerotic plaques and contribute to their progression. CAMs such as E-selectin, intercellular adhesion molecule-1 (ICAM-1), and vascular cell adhesion molecule-1 (VCAM-1) have been associated with the development of CVD in prospective cohorts [10–13]. For example, circulating levels of ICAM-1 were independently associated with incident CVD and carotid artery atherosclerosis (odds ratios of 5.53 and 2.64, respectively), and E-selectin was associated with carotid atherosclerosis (odds ratio = 2.03) [10]. OSA patients may have increased serum CAM levels as a result of endothelial dysfunction (a precursor to vascular disease) [14–16]. These molecules may thus represent useful biomarkers of CVD risk in OSA patients.

Genetic variants have been associated with circulating levels of CAMs in non-OSA cohorts. Two genome-wide association studies identified the *ABO* locus as the major genetic influence on soluble E-selectin (sE-selectin) levels [17, 18]. A study of sICAM-1 level [19] reported associations at the *ABO* and *ICAM1* loci [20, 21] and the *ABO* variants were the same as those associated with sE-selectin levels. In addition, four novel associations were identified in the *RELA* (RELA proto-oncogene, NF-κB subunit), *SH2B3* (SH2B adaptor protein 3), *NFKBIB* (NFκB inhibitor-β), and *PNPLA3* (patatin-like phospholipase domain containing 3) genes [19]. In contrast, there have been few studies of genetic factors that influence serum sVCAM-1 levels.

We hypothesized that genetic variants will be associated with plasma levels of soluble CAMs in patients suspected of OSA, and could partially explain the variability in the relationship between OSA and levels of these molecules. Furthermore, we hypothesized that there could be an interaction between these genetic markers and OSA to further increase levels of cell adhesion molecules. To investigate this, we measured CAM levels and identified single nucleotide polymorphisms (SNPs) that influence those levels in a sample of patients referred for suspected OSA.

## Materials and methods

### Study population

Adult patients with suspected OSA were recruited from the Sleep Apnea Clinical Research Registry (SACRR) at the University of British Columbia Hospital Sleep Disorders Program. Specifically, between 2003 and 2011, patients who were referred to the sleep clinic for suspected OSA and received an overnight diagnostic polysomnogram were recruited. Patients were excluded if they were medically unstable or had active psychiatric disease.

Weights and heights were measured and a comprehensive medical history questionnaire was completed. Patients were classified into three groups based on self-reported ethnicity: Caucasian, Southeast Asian/South Asian and Mixed/Other. Peripheral blood was collected by venipuncture the morning after the sleep study. All patients provided written informed consent and approval for the project was obtained from the University of British Columbia Research Ethics Board.

### Polysomnography

Attended inpatient polysomnography was performed in the hospital using standard techniques and involved measurements of electroencephalogram, electrocardiogram, oxygen saturation, airflow using nasal pressure, leg/chin electromyogram, eye movements, chest/abdominal excursion, and snoring. Polysomnograms including sleep stages and respiratory events were scored according to standard criteria [22]. The AHI was measured as follows: hypopneas (partial obstructions) were defined by ≥3% decrease in oxygen saturation and ≥30% decrease in nasal flow; apneas (total obstructions) were defined by ≥10 seconds of ≥90% decrease in airflow.

### Measurement of plasma proteins

Aliquots of blood, serum, and plasma were stored at -80°C. Samples of plasma were thawed and protein levels were measured using the MILLIPLEX MAP Human Cardiovascular Disease Panel 1 (HCVD1-67AK) multiplex Luminex bead assay (EMD Millipore, Etobicoke, ON, Canada). This technique enables the simultaneous measurement of several adhesion molecules including sE-selectin, sICAM-1, and sVCAM-1. The assays were performed according to the manufacturer's protocol using 25 μL of a 1:100 dilution of plasma, incubated overnight and tested in duplicate. The assay plate was read on a Luminex 100 System and the data were analyzed by Luminex 100 IS (Integrated System) Version 2.3.182 software. A standard curve was generated by the software using 5-parameter logistic curves of mean fluorescence intensity versus concentration.

### Polymorphism selection and genotyping

We selected polymorphisms previously shown to be associated with sE-selectin [17, 18] and sICAM1 [19, 20] levels. We also included polymorphisms (rs8176719 and rs8176746) to infer ABO blood types, as previously described [23]. Briefly, rs8176719 was used to identify the O blood group and rs8176746 was used to distinguish the A and B groups.

As there have been no comprehensive studies of the genetic control of sVCAM-1 levels, we surveyed the *VCAM1* gene for variants in Europeans and selected a subset of 13 tag SNPs that were representative of the genetic variation in this gene. This selection was performed using resequencing data in the European American Descent population of the SeattleSNPs Program for Genomic Applications (http://pga.mbt.washington.edu/) and the LDselect program [24]. LDselect parameter thresholds of linkage disequilibrium r^2^>0.8 and minor allele frequencies greater than 5% were used.

Genotyping was performed using the TaqMan method (Applied Biosystems, Foster City, CA). Genotyping quality control measures included the use of five DNA samples with known genotypes from the Centre d’Etudes du Polymorphime Humain (CEPH) panel as positive controls and eight no template wells as negative controls in each plate. In addition, 10% of the samples were genotyped in duplicate to test for reproducibility of the genotyping protocol. Hardy-Weinberg equilibrium tests were performed using the χ^2^ test with one degree of freedom and linkage disequilibrium estimation was performed using the CubeX cubic exact solutions program [25]. Polymorphisms were only retained in the analysis if the genotyping call rates were >99%.

### Statistical analysis

The major outcome variables were plasma levels of the three CAMs. These were natural log transformed to approximate a normal distribution. Univariate linear regression was used to determine the relationship between each SNP and relevant CAM levels, i.e., log (sE-selectin), log (sICAM-1), or log (sVCAM1). All tests of association between SNPs and the outcomes were performed under additive genetic models. Multivariate linear regressions were also performed controlling for the following confounders: age, gender, current smoking, AHI, previous coronary artery disease (self-report), and body mass index (BMI). Finally, we tested whether there was a significant interaction between AHI and the relevant SNP. All statistical analyses were conducted using SAS version 9.2 (SAS Institute, Cary, NC).

## Results

### Patient characteristics

The characteristics of the 491 study subjects are shown in Table 1. The mean age was 49.7 years and the majority (68%) were male, reflecting a typical population with suspected OSA [1]. Following polysomnography, 23.7% were identified as not having OSA (AHI <5/hr) and 24.7% had severe OSA (AHI ≥30/hr). The summary statistics for the plasma CAM levels are shown in Table A in S1 File.

**Table 1:** Distribution of demographic and clinical characteristics for subjects in the Sleep Apnea Clinical Research Registry cohort.

Characteristic		Mean ± SD	N
Age, years		49.7 ± 11.8	488
Body mass index, kg/m^2^		31.8 ± 6.7	483
Apnea hypopnea index		22.6 ± 21.7	491
Sex	Men		337 (69.9%)
	Women		153 (30.9%)
Smoking history	Smoker		43 (12.4%)
	Non-smoker		303 (87.6%)
Ethnic group	Caucasian		407 (78.8%)
	Asian/ South Asian		45 (8.8%)
	Other/ Mixed		58 (11.4%)

### Genotyping

The individuals in the SACRR cohort were genotyped for 24 polymorphisms. Four polymorphisms were omitted from the analysis as the genotyping assays failed (rs3176878 and rs3917012 in *VCAM1*) or call rates were low (rs3184504 in *SH2B3* and rs3176867 in *VCAM1*). All of the remaining 20 polymorphisms were in Hardy-Weinberg equilibrium (Table B in S1 File). The linkage disequilibrium (LD) between SNPs in the same gene is shown in Table C in S1 File. As expected, none of the *VCAM1* polymorphisms were in strong LD (i.e. r^2^<0.8 for all pairs of SNPs).

### Soluble E-selectin level

In univariate analyses, the *ABO* rs579459 SNP and ABO blood groups were significantly associated with log(sE-selectin) levels (Table 2). Specifically, the minor allele C of rs579459 was associated with lower sE-selectin levels, in agreement with previous studies [17, 18]. rs579459 was a more significant predictor of log(sE-selectin) levels (R^2^ = 0.165, *P* = 7 × 10^−21^) than ABO blood group (R^2^ = 0.129, *P* = 2 × 10^−14^). Similar results were observed in Caucasian patients only (Table D in S1 File). The rs579459 polymorphism and ABO blood groups were associated with log(sE-selectin) levels in both OSA and non-OSA individuals (Table E in S1 File).

**Table 2:** Association of *ABO* genotype and blood group with log(soluble E-selectin) levels.

SNP / blood group	Genotype / blood group	N	Mean (± SD) log(soluble E-selectin) level	R^2^	*P* value*
rs579459	TT	302	3.908 ± 0.360	0.165	7 × 10^−21^
	TC	171	3.563 ± 0.400		
	CC	16	3.397 ± 0.776		
ABO blood group	A	206	3.604 ± 0.455	0.129	2 × 10^−14^
	AB	19	3.624 ± 0.447		
	B	56	3.841 ± 0.362		
	O	208	3.930 ± 0.353		

*Analyzed by linear regression under an additive genetic model for rs579459 and by one-way ANOVA for ABO blood group

In a multivariate analysis, several variables were significantly associated with log(sE-selectin) including sleep apnea severity, BMI, age and gender (Table 3). rs579459 was significantly associated with E-selectin levels when added to the model. Furthermore, the goodness of fit of the model was improved as indicated by a reduction in the Akaike information criterion (AIC), suggesting that the SNP information explained an additional proportion of the variability in log(sE-selectin) level (Table 3).

**Table 3:** Multivariate associations with log(soluble E-selectin) levels.

Variable	Model 1*		Model 2^†^	
	Estimate	*P* value	Estimate	*P* value
Apnea hypopnea index	0.0024	0.080	0.0015	0.067
Preexisting heart disease	0.038	0.82	0.037	0.71
Male gender	0.16	<0.0001	0.13	0.0003
Age	-0.0032	0.0016	-0.0025	0.082
Body mass index	0.02	0.0029	0.019	<0.0001
Current smoking	0.092	0.16	0.081	0.16
Ethnic Group: Asian / South Asian *vs*. Caucasian	0.037	0.56	0.029	0.62
Ethnic Group: Other / Mixed vs. Caucasian	0.11	0 .058	0.11	0.037
rs579459 (number of T alleles)	-	-	0.29	<0.0001
Akaike information criterion	456		373	

*Multivariate linear regression controlling for age, gender, current smoking, apnea hypopnea index, previous coronary artery disease (self-report), ethnic group and body mass index.

^**†**^Multivariate linear regression controlling for the variables in Model 1 plus rs579459.

We determined whether rs579459, and polymorphisms in high LD with it, have been associated with gene expression. Several of these SNPs were significantly associated with *ABO* gene expression as well as the expression of other genes in the same chromosomal region (Table F in S1 File). The SNPs were also associated with several protein and metabolite levels (Table G in S1 File).

### Soluble ICAM-1 level

We investigated eight polymorphisms previously shown to be associated with sICAM-1 levels (Table 4). Of these, only rs1799969 and rs5498 in *ICAM1* and rs738409 in *PNPLA3* were associated in the SACRR patients. The direction of these associations was the same as the previous literature [19, 20] but the proportion of the variance explained was much lower in the SACRR subjects. Furthermore, these associations must be interpreted with caution, as they were no longer significant after Bonferroni correction for multiple comparisons (significance threshold required to correct for testing of 20 SNPs, *P* <0.05/20 = 0.0025). Analyses stratified by OSA status are shown in Table H in S1 File.

**Table 4:** Association of genotypes with log(soluble ICAM-1) levels.

Gene	SNP	Genotype	N	Mean (± SD) log(soluble ICAM1) level	R^2^	*P* value
*ABO*	rs579459	TT	298	4.232 ± 0.499	0.006	0.086
		TC	170	4.130 ± 0.501		
		CC	16	4.217 ± 0.392		
*ICAM1*	rs11575074	GG	426	4.199 ± 0.489	0.000	0.674
		GA	58	4.170 ± 0.566		
	rs1799969	GG	387	4.214 ± 0.517	0.011	**0.018**
		GA	88	4.165 ± 0.381		
		AA	9	3.694 ± 0.439		
	rs5498	AA	148	4.116 ± 0.518	0.011	**0.020**
		AG	227	4.222 ± 0.496		
		GG	106	4.257 ± 0.463		
	rs281438	TT	247	4.226 ± 0.503	0.003	0.219
		TG	198	4.162 ± 0.479		
		GG	39	4.171 ± 0.556		
*NFKBIB*	rs3136642	AA	177	4.180 ± 0.510	0.000	0.752
		AG	217	4.209 ± 0.505		
		GG	90	4.193 ± 0.462		
*PNPLA3*	rs738409	CC	267	4.166 ± 0.508	0.011	**0.020**
		CG	189	4.198 ± 0.478		
		GG	28	4.463 ± 0.472		
*RELA*	rs1049728	GG	431	4.194 ± 0.495	0.000	0.884
		GC	51	4.218 ± 0.534		
		CC	2	4.039		

In the multivariate analysis, BMI, current smoking status, ethnicity and previous coronary artery disease were associated with log(sICAM-1) levels. When rs1799969 was added to the model, the A allele was associated with a decrease in ICAM1 levels (*P* = 0.0061) and there was a reduction in AIC (696 *vs*. 690). The G allele of rs738409 in *PNPLA3* was associated with increased ICAM1 levels (*P* = 0.0099). The model was improved with addition of the rs738409 genotype information (AIC = 691).

rs1799969 and rs5498 have been associated with the expression of multiple ICAM genes but most significantly with *ICAM4* (Table I in S1 File). rs738409 has not been associated with ICAM gene expression but was modestly associated with *SAMM50* expression (a gene located close to *PNPLA3* (Table I in S1 File).

### Soluble VCAM-1 level

We investigated 10 *VCAM1* polymorphisms but none of them were associated with log(VCAM-1) plasma protein levels in the univariate analyses (Table 5). In the multivariate analysis, log(sVCAM-1) level was not associated with AHI in the SACRR patients but was significantly associated with BMI, age, and gender (*P*<0.05, AIC = -68.9). When rs3176874 was added to the model, it was associated with log(sVCAM-1) levels (p = 0.022) although the AIC did not reduce substantially (-68.6). rs3176877 was not significant when added to the model. Analyses stratified by OSA status are shown in Table J in S1 File.

**Table 5:** Association of *VCAM1* genotypes with log(soluble VCAM-1) levels.

SNP	Genotype	N	Mean (± SD) log(soluble VCAM1 level	R^2^	*P* value
rs1582091	GG	121	6.839 ± 0.222	0.006	0.080
	GT	254	6.787 ± 0.213		
	TT	114	6.785 ± 0.296		
rs3176860	AA	164	6.829 ± 0.218	0.003	0.203
	AG	245	6.779 ± 0.219		
	GG	80	6.802 ± 0.315		
rs3176861	CC	293	6.807 ± 0.238	0.001	0.451
	CT	168	6.786 ± 0.228		
	TT	26	6.797 ± 0.300		
rs3176863	GG	342	6.804 ± 0.232	0.000	0.619
	GA	134	6.788 ± 0.222		
	AA	12	6.808 ± 0.487		
rs3176869	AA	356	6.796 ± 0.224	0.000	0.720
	AT	123	6.814 ± 0.277		
	TT	5	6.712 ± 0.142		
rs3176874	AA	369	6.790 ± 0.235	0.007	0.072
	AG	109	6.824 ± 0.244		
	GG	10	6.894 ± 0.257		
rs3176877	TT	181	6.780 ± 0.236	0.008	0.050
	TA	239	6.798 ± 0.243		
	AA	65	6.853 ± 0.219		
rs3181088	CC	346	6.793 ± 0.241	0.004	0.153
	CT	130	6.806 ± 0.217		
	TT	13	6.915 ± 0.311		
rs3917009	CC	419	6.799 ± 0.237	0.000	0.834
	CT	68	6.800 ± 0.245		
	TT	2	6.892		
rs6660837	CC	259	6.794 ± 0.255	0.000	0.692
	AC	189	6.807 ± 0.223		
	AA	38	6.797 ± 0.180		

### Joint effect of polymorphisms and AHI on plasma protein levels

For sE-selectin levels, there appeared to be an interaction between the presence of the rs579459 TT genotype and OSA (Table 6). Specifically, severe OSA was associated with a larger change in log(sE-selectin) levels in patients who did not have the TT polymorphism (*P* = 0.05 test for interaction). However, the combination of both factors (i.e. OSA and a deleterious genotype) was associated with the highest levels of sE-selectin.

**Table 6:** Joint effect of rs579459 and AHI on soluble E-selectin levels.

rs579459 genotype	Apnea Hypopnea Index	N	Mean (± SD) soluble E-selectin level (ng/ml)
CC/TC	<30/hr	141	35.6 ± 13.6
CC/TC	>30/hr	44	49.0 ± 33.6
TT	<30/hr	216	52.8 ± 22.0
TT	>30/hr	85	54.8 ± 17.0

## Discussion

In our cohort of patients with suspected OSA, we found significant associations between various SNPs and levels of CAMs. This was especially robust for sE-selectin, where the T allele of rs579459 was associated with increased levels. Both sleep apnea severity (as assessed by AHI) and rs579459 were significantly correlated with sE-selectin levels, and there was an interaction between these two variables. Specifically, the increase in sE-selectin levels associated with severe OSA was greater in patients without the TT genotype, although the combination of severe OSA and the TT polymorphism identified a group of patients with high sE-selectin levels. Given the relationship between sE-selectin levels and cardiac events, rs579459 may help to identify patients at greatest risk of incident CVD. Ironically, the impact of OSA treatment might be greater in patients without the TT genotype. Nevertheless, the interaction between the rs579459 TT genotype and severe OSA was of borderline statistical significance and therefore should be viewed with caution until verified in another population.

rs579459 accounted for 16% of the variance in sE-selectin levels (Table 2), which is consistent with the estimate (19%) in a study of Caucasian type 1 diabetes patients [17]. Another study of Caucasian participants [18] found the strongest association with rs651007, which is in perfect LD with rs579459 in the European population. These previous observations and our own data showing highly significant association of rs579459 in Caucasians suggest that our results are not due to population stratification. However, despite the strong statistical association, rs579459 is not strongly predictive of sE-selectin level.

Since variation in the *ABO* gene is associated with sE-selectin levels, a possible underlying mechanism is that the ABO blood groups are the causal factors. The *ABO* gene encodes a glycosyltransferase that catalyzes the transfer of monosaccharides to the precursor H antigen. The A and B alleles encode transferases that differ by four amino acids [26] and have different substrate specificities, whereas the O allele contains a frame-shift deletion that results in an inactive enzyme. Thus, one potential mechanism to explain the association of *ABO* variants and sE-selectin levels could be the lack of glycosyltransferase activity in individuals of the O blood type. In our data and in two published studies [18, 27] there is a consistent trend in sE-selectin levels stratified by the main ABO blood groups (Figure A in S1 File) with individuals of the O type having the highest levels.

The association of ABO polymorphisms with sE-selectin may not be solely related to ABO blood types. We and others [17] have shown that rs579459 is a more significant predictor of sE-selectin levels than ABO blood groups, suggesting that rs579459 (or another variant in LD with it) is contributing to plasma levels. rs579459 is located in the 5’ region of the *ABO* gene and is in high LD with several other variants, many of which have been associated with the expression of *ABO* and other genes (Table F in S1 File). However, these variants have been associated with various blood protein levels and related traits (Table G in S1 File) making the underlying mechanism for the association unclear.

Soluble E-selectin, ICAM-1 and VCAM-1 are present in the blood due to shedding or proteolytic cleavage of the membrane-bound forms from the endothelium [28, 29]. sICAM-1 inhibits leukocyte adhesion to the endothelium [30] and other soluble CAMs may function in a similar manner, directly contributing to CVD risk. Alternatively, soluble CAM levels may simply reflect the amount of the corresponding membrane-bound forms [28]. ICAM-1 is known to be glycosylated [31] and the level of glycosylation may affect cleavage into the soluble form or clearance. There is evidence to suggest that the ABO glycosylation contributes to von Willebrand factor levels by protection from proteolysis [32, 33] and affecting the clearance rate [34].

We have shown that the minor allele (C) of rs579459 was associated with lower sE-selectin levels. Elevated levels of sE-selectin are associated with increased risk of CVD [10]. Paradoxically, the minor allele of rs579459 was associated with increased risk of coronary artery disease [35], venous thromboembolism [36], and myocardial infarction [37]. Similarly, non-O blood groups were associated with lower sE-selectin levels but increased risk of venous thromboembolism [38].

There are potential limitations to our study. First, we only studied a modest number of patients in one sleep centre and the generalizability our findings to other populations (especially different ethnic groups) is still to be determined. However, the relationships we found between SNPs and levels of adhesion molecules are consistent with other studies [17, 18]. Second, we did not study clinical events such as incident myocardial infarction or stroke. Future studies are needed to determine whether CAM levels predict events in this population and whether these polymorphisms are helpful in prognostication. However, several of the polymorphisms in this study have been associated with CVD outcomes (Table K in S1 File). Third, we measured the levels of circulating CAMs and these may not accurately represent the level of the membrane-bound forms, which may be more physiologically relevant. Lastly, we do not know if intermittent or continuous hypoxia was a more important factor in our results. However, given that the majority of these patients had OSA, we suspect that intermittent hypoxia is likely the key factor. Nevertheless, we believe these results are relevant in terms of eventually developing a more precision-based approach to the care of patients with OSA using genetic biomarkers.

## Supporting information

S1 File Tables A-K and Figure A. (PDF)
