# Supplementary material for: Adhesion molecule gene variants and plasma protein levels in patients with suspected obstructive sleep apnea
Source: PLoS One. 2019 Jan 17;14(1):e0210732. doi: 10.1371/journal.pone.0210732 (PMC6336279; doi:10.1371/journal.pone.0210732)
Supplement: S1 File — (PDF) [file pone.0210732.s001.pdf]

# Supporting information

**Adhesion Molecule Gene Variants and Plasma Protein Levels in Patients with Suspected OSA**

Andrew J Sandford, Amanda Ha, David A Ngan, Loubna Akhabir, Aabida Saferali, Nurit Fox, AJ Hirsch Allen, Simon C Warby, Stephan VanEeden, Najib T Ayas

**Table A.** Plasma protein levels for subjects in the Sleep Apnea Clinical Research Registry.

| **Plasma protein** | **Mean** | **SD** | **N** |
|---|---|---|---|
| E-Selectin (ng/mL) | 47.6 | 21.6 | 489 |
| sICAM-1 (ng/mL) | 74.1 | 34.6 | 484 |
| sVCAM-1 (ng/mL) | 922.5 | 218.0 | 489 |

**Table B.** Genotype distributions of single nucleotide polymorphisms and assessment of Hardy-Weinberg equilibrium (HWE) in the Sleep Apnea Clinical Research Registry patients.

| Gene | Polymorphism | Genotype | N (%) | HWE *P* value | N (%) Caucasians | HWE *P* value Caucasians |
|---|---|---|---|---|---|---|
| *ABO* | rs579459 | TT | 323 (62) | 0.154 | 246 (61) | 0.421 |
| | | TC | 182 (35) | | 142 (35) | |
| | | CC | 17 (3) | | 16 (4) | |
| | rs8176719 | -\|- | 224 (43) | 0.599 | 170 (42) | 0.581 |
| | | -\|C | 240 (46) | | 188 (46) | |
| | | C\|C | 58 (11) | | 46 (11) | |
| | rs8176746 | GG | 441 (84) | 0.399 | 349 (86) | 0.493 |
| | | GT | 76 (15) | | 52 (13) | |
| | | TT | 5 (1) | | 3 (1) | |
| *ICAM1* | rs11575074 | GG | 459 (88) | 0.142 | 354 (88) | 0.185 |
| | | GA | 63 (12) | | 50 (12) | |
| | | AA | 0 | | 0 | |
| | rs1799969 | GG | 414 (79) | 0.146 | 313 (77) | 0.093 |
| | | GA | 98 (19) | | 81 (20) | |
| | | AA | 10 (2) | | 10 (2) | |
| | rs5498 | AA | 160 (31) | 0.363 | 121 (30) | 0.696 |
| | | AG | 247 (48) | | 195 (49) | |
| | | GG | 112 (22) | | 85 (21) | |
| | rs281438 | TT | 272 (52) | 0.986 | 198 (49) | 0.823 |
| | | TG | 209 (40) | | 171 (42) | |
| | | GG | 40 (8) | | 35 (9) | |
| *NFKBIB* | rs3136642 | AA | 190 (36) | 0.164 | 160 (40) | 0.467 |
| | | AG | 237 (45) | | 183 (45) | |
| | | GG | 95 (18) | | 61 (15) | |
| *PNPLA3* | rs738409 | CC | 289 (55) | 0.468 | 232 (58) | 0.324 |
| | | CG | 203 (39) | | 153 (38) | |
| | | GG | 30 (6) | | 19 (5) | |

| | | | | | | |
|---|---|---|---|---|---|---|
| *RELA* | rs1049728 | GG | 464 (89) | 0.824 | 356 (88) | 0.698 |
| | | GC | 56 (11) | | 46 (11) | |
| | | CC | 2 (0) | | 2 (0) | |
| *VCAM1* | rs1582091 | GG | 128 (24) | 0.334 | 99 (25) | 0.107 |
| | | GT | 272 (52) | | 218 (55) | |
| | | TT | 122 (23) | | 87 (21) | |
| | rs3176860 | AA | 171 (33) | 0.277 | 140 (35) | 0.201 |
| | | AG | 266 (51) | | 206 (51) | |
| | | GG | 85 (16) | | 58 (14) | |
| | rs3176861 | CC | 307 (59) | 0.985 | 249 (62) | 0.917 |
| | | CT | 185 (36) | | 136 (34) | |
| | | TT | 28 (5) | | 18 (4) | |
| | rs3176863 | GG | 364 (70) | 0.582 | 282 (70) | 0.585 |
| | | GA | 145 (28) | | 112 (28) | |
| | | AA | 12 (2) | | 9 (2) | |
| | rs3176869 | AA | 381 (74) | 0.085 | 292 (73) | 0.049 |
| | | AT | 131 (25) | | 105 (26) | |
| | | TT | 5 (1) | | 3 (1) | |
| | rs3176874 | AA | 396 (76) | 0.593 | 310 (77) | 0.676 |
| | | AG | 114 (22) | | 85 (21) | |
| | | GG | 10 (2) | | 7 (2) | |
| | rs3176877 | TT | 198 (38) | 0.450 | 166 (42) | 0.848 |
| | | TA | 251 (48) | | 182 (46) | |
| | | AA | 69 (13) | | 52 (13) | |
| | rs3181088 | CC | 366 (70) | 0.560 | 269 (67) | 0.723 |
| | | CT | 140 (27) | | 120 (30) | |
| | | TT | 16 (3) | | 15 (4) | |
| | rs3917009 | CC | 450 (86) | 0.679 | 339 (84) | 0.611 |
| | | CT | 70 (13) | | 63 (16) | |
| | | TT | 2 (0) | | 2 (0) | |
| | rs6660837 | CC | 276 (53) | 0.635 | 212 (53) | 0.731 |
| | | AC | 202 (39) | | 161 (40) | |
| | | AA | 41 (8) | | 28 (7) | |

**Table C.** Linkage disequilibrium (LD) of single nucleotide polymorphisms in the Sleep Apnea Clinical Research Registry patients. The LD is expressed as two metrics: $r^2$ above the diagonal and D' below the diagonal.

| **ABO** | rs579459 | rs8176719 | rs8176746 |
|---|---|---|---|
| rs579459 | | 0.4796 | 0.0234 |
| rs8176719 | 0.975 | | 0.1653 |
| rs8176746 | 1 | 0.976 | |

| **ICAM1** | rs11575074 | rs1799969 | rs5498 | rs281438 |
|---|---|---|---|---|
| rs11575074 | | 0.0014 | 0.0335 | 0.0934 |
| rs1799969 | 0.419 | | 0.1411 | 0.0358 |
| rs5498 | 0.79 | 0.956 | | 0.2974 |
| rs281438 | 0.746 | 0.854 | 0.969 | |

| **VCAM1** | rs1582091 | rs3176860 | rs3176861 | rs3176863 | rs3176869 | rs3176874 | rs3176877 | rs3181088 | rs3917009 | rs6660837 |
|---|---|---|---|---|---|---|---|---|---|---|
| rs1582091 | | 0.6907 | 0.2765 | 0.1981 | 0.0019 | 0.0007 | 0.0014 | 0.0001 | 0.0642 | 0.0016 |
| rs3176860 | 0.97 | | 0.3845 | 0.2544 | 0 | 0.0004 | 0.0009 | 0.0017 | 0.0547 | 0.0023 |
| rs3176861 | 0.945 | 0.955 | | 0.0584 | 0.0238 | 0.0014 | 0.0013 | 0.0076 | 0.0058 | 0.0001 |
| rs3176863 | 1 | 0.972 | 1 | | 0.0107 | 0 | 0.0003 | 0.0022 | 0.0146 | 0.006 |
| rs3176869 | 0.109 | 0.003 | 0.212 | 0.59 | | 0.0037 | 0.0053 | 0.0096 | 0.0069 | 0.0014 |
| rs3176874 | 0.066 | 0.06 | 0.177 | 0.035 | 0.063 | | 0.1794 | 0.0291 | 0.0125 | 0.0026 |
| rs3176877 | 0.047 | 0.032 | 0.051 | 0.049 | 0.239 | 0.853 | | 0.1089 | 0.0005 | 0.0002 |
| rs3181088 | 0.025 | 0.108 | 0.356 | 0.048 | 0.11 | 1 | 0.967 | | 0.0021 | 0.052 |
| rs3917009 | 0.907 | 1 | 0.503 | 1 | 0.121 | 0.155 | 0.06 | 0.074 | | 0.0001 |
| rs6660837 | 0.065 | 0.093 | 0.008 | 0.286 | 0.154 | 0.217 | 0.027 | 0.837 | 0.026 | |

**Table D.** Association of *ABO* genotype and blood group with soluble E-selectin levels in Caucasian patients.

| SNP / blood group | Genotype / blood group | N | Mean (± SD) $log_{10}$(soluble E-selectin) level | $R^2$ | *P* value |
|---|---|---|---|---|---|
| rs579459 | TT | 234 | 3.898 ± 0.355 | 0.180 | $5 \times 10^{-18}$ |
| | TC | 131 | 3.553 ± 0.374 | | |
| | CC | 15 | 3.352 ± 0.782 | | |
| ABO blood group | A | 168 | 3.610 ± 0.452 | 0.125 | $7 \times 10^{-11}$ |
| | AB | 13 | 3.505 ± 0.368 | | |
| | B | 38 | 3.853 ± 0.370 | | |
| | O | 161 | 3.910 ± 0.347 | | |

**Table E.** Analysis of *ABO* rs579459 genotype and log(soluble E-selectin) levels stratified by obstructive sleep apnea status.

| SNP / blood group | Genotype / blood group | Obstructive sleep apnea | | | | No obstructive sleep apnea | | | |
|---|---|---|---|---|---|---|---|---|---|
| | | N | Mean (± SD) log(soluble E-selectin) level | $R^2$ | *P* value | N | Mean (± SD) log(soluble E-selectin) level | $R^2$ | *P* value |
| rs579459 | TT | 238 | 3.944 ± 0.354 | 0.163 | <0.0001 | 65 | 3.875 ± 0.342 | 0.220 | <0.0001 |
| | TC | 131 | 3.602 ± 0.384 | | | 40 | 3.563 ± 0.404 | | |
| | CC | 13 | 3.540 ± 0.767 | | | 3 | 2.994 ± 0.600 | | |
| ABO blood group | A | 160 | 3.650 ± 0.444 | 0.121 | <0.0001 | 46 | 3.576 ± 0.431 | 0.160 | 0.0004 |
| | AB | 14 | 3.722 ± 0.401 | | | 5 | 3.458 ± 0.518 | | |
| | B | 42 | 3.883 ± 0.384 | | | 14 | 3.806 ± 0.238 | | |
| | O | 166 | 3.959 ± 0.341 | | | 43 | 3.914 ± 0.362 | | |

**Table F.** Association of *ABO* polymorphisms with gene expression. Data accessed via HaploReg v4.1 (http://archive.broadinstitute.org/mammals/haploreg/haploreg.php).

| Polymorphism | Chromosomal position (hg38) | LD ($r^2$) with rs579459* | Location | Tissue | Correlated gene | *P* value | Reference |
|---|---|---|---|---|---|---|---|
| rs2519093 | Chr9: 133,266,456 | 0.823 | *ABO* intron 1 | Whole blood | *ABO* | $7 \times 10^{-09}$ | [1] |
| | | | | Lymphoblastoid cell line | *SURF1* | $7 \times 10^{-08}$ | [2] |
| | | | | Adrenal gland | *ABO* | $4 \times 10^{-06}$ | [1] |
| | | | | Heart - atrium | *SURF1* | $4 \times 10^{-06}$ | [1] |
| rs507666 | Chr9: 133,273,983 | 0.828 | *ABO* intron 1 | Whole blood | *ABO* | $3 \times 10^{-09}$ | [1] |
| | | | | Lymphoblastoid cell line | *SURF1* | $8 \times 10^{-08}$ | [2] |
| | | | | Adrenal gland | *ABO* | $3 \times 10^{-06}$ | [1] |
| rs8176643 | Chr9: 133,274,293 | 0.817 | *ABO* intron 1 | Whole blood | *ABO* | $4 \times 10^{-08}$ | [1] |
| | | | | Adrenal gland | *ABO* | $3 \times 10^{-06}$ | [1] |
| rs532436 | Chr9: 133,274,414 | 0.828 | *ABO* intron 1 | Whole blood | *ABO* | $3 \times 10^{-09}$ | [1] |
| | | | | Lymphoblastoid cell line | *SURF1* | $1 \times 10^{-07}$ | [2] |
| | | | | Adrenal gland | *ABO* | $3 \times 10^{-06}$ | [1] |
| rs600038 | Chr9: 133,276,354 | 0.994 | 1.1 kb 5' of *ABO* | Whole blood | *ABO* | $3 \times 10^{-09}$ | [1] |
| | | | | Lymphoblastoid cell line | *SURF1* | $2 \times 10^{-06}$ | [2] |
| rs651007 | Chr9: 133,278,431 | 1 | 3.2 kb 5' of *ABO* | Whole blood | *ABO* | $8 \times 10^{-10}$ | [1] |
| | | | | Whole blood | *SURF6* | $8 \times 10^{-07}$ | [3] |
| | | | | Lymphoblastoid cell line | *SURF1* | $1 \times 10^{-06}$ | [2] |
| | | | | Whole blood | *GBGT1* | $6 \times 10^{-06}$ | [3] |
| | | | | Whole blood | *SURF4* | $4 \times 10^{-04}$ | [3] |
| rs579459 | Chr9: 133,278,724 | - | 3.5 kb 5' of *ABO* | Whole blood | *ABO* | $8 \times 10^{-10}$ | [1] |
| | | | | Lymphoblastoid cell line | *SURF1* | $1 \times 10^{-06}$ | [2] |
| | | | | Whole blood | *GBGT1* | $2 \times 10^{-06}$ | [3] |
| | | | | Whole blood | *SURF6* | $2 \times 10^{-05}$ | [3] |
| | | | | Whole blood | *SURF4* | $2 \times 10^{-04}$ | [3] |

| **Polymorphism** | **Chromosomal position (hg38)** | **LD ($r^2$) with rs579459*** | **Location** | **Tissue** | **Correlated gene** | ***P* value** | **Reference** |
|---|---|---|---|---|---|---|---|
| rs649129 | Chr9: 133,278,860 | 1 | 3.6 kb 5' of *ABO* | Whole blood | *ABO* | $8 \times 10^{-10}$ | [1] |
| | | | | Lymphoblastoid cell line | *SURF1* | $1 \times 10^{-06}$ | [2] |
| | | | | Whole blood | *GBGT1* | $2 \times 10^{-06}$ | [3] |
| | | | | Whole blood | *SURF6* | $3 \times 10^{-05}$ | [3] |
| | | | | Whole blood | *SURF4* | $2 \times 10^{-04}$ | [3] |
| rs495828 | Chr9: 133,279,294 | 1 | 4.1 kb 5' of *ABO* | Whole blood | *ABO* | $8 \times 10^{-10}$ | [1] |
| | | | | Lymphoblastoid cell line | *SURF1* | $2 \times 10^{-06}$ | [2] |
| | | | | Whole blood | *GBGT1* | $2 \times 10^{-06}$ | [3] |
| | | | | Whole blood | *SURF6* | $3 \times 10^{-05}$ | [3] |
| | | | | Whole blood | *SURF4* | $2 \times 10^{-04}$ | [3] |
| rs635634 | Chr9: 133,279,427 | 0.834 | 4.2 kb 5' of *ABO* | Whole blood | *ABO* | $6 \times 10^{-10}$ | [1] |
| | | | | Lymphoblastoid cell line | *SURF1* | $1 \times 10^{-07}$ | [2] |
| | | | | Adrenal gland | *ABO* | $3 \times 10^{-06}$ | [1] |

*Data from 1000 Genomes Project Phase 3: EUR population (n=503)

**Table G.** Association of *ABO* polymorphisms with protein levels and other traits. Data accessed via HaploReg v4.1 (http://archive.broadinstitute.org/mammals/haploreg/haploreg.php).

| Polymorphism | Chromosomal position (hg38) | LD ($r^2$) with rs579459* | Location | Tissue | Correlated trait | *P* value | Reference |
|---|---|---|---|---|---|---|---|
| rs507666 | Chr9: 133,273,983 | 0.828 | *ABO* intron 1 | Plasma | Soluble ICAM-1 level | $3 \times 10^{-91}$ | [4] |
| | | | | Plasma | Soluble ICAM-1 level | $5 \times 10^{-29}$ | [5] |
| | | | | Plasma | Low-density lipoprotein cholesterol level | $2 \times 10^{-11}$ | [6] |
| | | | | Plasma | Total cholesterol level | $4 \times 10^{-11}$ | [6] |
| rs651007 | Chr9: 133,278,431 | 1 | 3.2 kb 5' of *ABO* | Plasma | Soluble E-selectin level | $2 \times 10^{-82}$ | [7] |
| | | | | Serum | Alkaline phosphatase level | $1 \times 10^{-56}$ | [8] |
| | | | | Blood | Factor VIII level | $2 \times 10^{-25}$ | [9] |
| | | | | Serum | ADpSGEGDFXAEGGGVR† level | $9 \times 10^{-19}$ | [10] |
| | | | | Plasma | Low-density lipoprotein cholesterol level | $6 \times 10^{-9}$ | [11] |
| | | | | Serum | Ferritin level | $1 \times 10^{-8}$ | [12] |
| rs579459 | Chr9: 133,278,724 | - | 3.5 kb 5' of *ABO* | Serum | Soluble E-selectin level | $1 \times 10^{-29}$ | [13] |
| | | | | Plasma | Soluble P-selectin level | $2 \times 10^{-41}$ | [14] |
| | | | | Plasma | Alkaline phosphatase level | $3 \times 10^{-123}$ | [15] |
| | | | | Blood | Red blood cell count | $9 \times 10^{-18}$ | [16] |
| | | | | Urine | Unknown metabolite level | $2 \times 10^{-32}$ | [17] |
| | | | | Urine | Unknown metabolite level | $1 \times 10^{-28}$ | [17] |
| | | | | Serum | ADpSGEGDFXAEGGGVR†/X-14304—leucylalanine ratio | $1 \times 10^{-28}$ | [10] |
| rs649129 | Chr9: 133,278,860 | 1 | 3.6 kb 5' of *ABO* | Plasma | Soluble ICAM-1 level | $1 \times 10^{-15}$ | [14] |
| | | | | Serum | ADSGEGDFXAEGGGVR‡/ADpSGEGDFXAEGGGVR† ratio | $9 \times 10^{-37}$ | [10] |

| | Chromosomal position (hg38) | LD ($r^2$) with rs579459* | Location | Tissue | Correlated trait | P value | Reference |
|---|---|---|---|---|---|---|---|
| rs495828 | Chr9: 133,279,294 | 1 | 4.1 kb 5' of *ABO* | Plasma | Angiotensin-converting enzyme activity | $3 \times 10^{-8}$ | [18] |
| | | | | Serum | Alkaline phosphatase level | $4 \times 10^{-59}$ | [19] |
| | | | | Blood | Red blood cell count | $3 \times 10^{-12}$ | [19] |
| | | | | Blood | Hematocrit | $1 \times 10^{-11}$ | [19] |
| | | | | Blood | Hematological biochemical traits | $6 \times 10^{-10}$ | [19] |
| | | | | Serum | DSGEGDFXAEGGGVR[||]/ADpSGEGDFXAE GGGVR[†] ratio | $6 \times 10^{-34}$ | [10] |
| rs635634 | Chr9: 133,279,427 | 0.834 | 4.2 kb 5' of *ABO* | Plasma | Low-density lipoprotein cholesterol level | $8 \times 10^{-22}$ | [20] |
| | | | | Plasma | Cholesterol total | $9 \times 10^{-21}$ | [20] |

*Data from 1000 Genomes Phase 3: EUR population (n=503)

†Alanyl-α-aspartyl-O-phosphonoserylglycyl-α-glutamylglycyl-α-aspartylphenylalanylleucylalanyl-α-glutamylglycylglycylglycylvalylarginine

‡Alanyl-α-aspartylserylglycyl-α-glutamylglycyl-α-aspartylphenylalanylleucylalanyl-α-glutamylglycylglycylglycylvalylarginine

||α-Aspartylserylglycyl-α-glutamylglycyl-α-aspartylphenylalanylleucylalanyl-α-glutamylglycylglycylglycylvalylarginine

**Table H.** Analysis of genotypes with log(soluble ICAM-1) levels stratified by obstructive sleep apnea status.

| SNP | Genotype | Obstructive sleep apnea | | | | No obstructive sleep apnea | | | |
|---|---|---|---|---|---|---|---|---|---|
| | | N | Mean (± SD) log(soluble ICAM1) level | $R^2$ | *P* value | N | Mean (± SD) log(soluble ICAM1) level | $R^2$ | *P* value |
| rs579459 | TT | 238 | 4.217 ± 0.547 | 0.007 | 0.279 | 65 | 4.240 ± 0.512 | 0.005 | 0.771 |
| | TC | 131 | 4.129 ± 0.492 | | | 40 | 4.164 ± 0.567 | | |
| | CC | 13 | 4.248 ± 0.345 | | | 3 | 4.166 ± 0.620 | | |
| rs11575074 | GG | 337 | 4.201 ± 0.492 | 0.005 | 0.156 | 92 | 4.228 ± 0.514 | 0.006 | 0.414 |
| | GA | 45 | 4.084 ± 0.717 | | | 16 | 4.109 ± 0.631 | | |
| rs1799969 | GG | 302 | 4.216 ± 0.529 | 0.021 | 0.016 | 88 | 4.240 ± 0.506 | 0.021 | 0.326 |
| | GA | 72 | 4.119 ± 0.479 | | | 19 | 4.103 ± 0.636 | | |
| | AA | 8 | 3.732 ± 0.455 | | | 1 | 3.626 | | |
| rs5498 | AA | 114 | 4.069 ± 0.580 | 0.022 | 0.015 | 36 | 4.236 ± 0.415 | 0.022 | 0.316 |
| | AG | 184 | 4.230 ± 0.508 | | | 47 | 4.144 ± 0.627 | | |
| | GG | 83 | 4.252 ± 0.451 | | | 23 | 4.347 ± 0.466 | | |
| rs281438 | TT | 201 | 4.205 ± 0.526 | 0.002 | 0.650 | 49 | 4.318 ± 0.502 | 0.067 | 0.026 |
| | TG | 153 | 4.178 ± 0.510 | | | 48 | 4.059 ± 0.564 | | |
| | GG | 28 | 4.111 ± 0.585 | | | 11 | 4.389 ± 0.335 | | |
| rs3136642 | AA | 140 | 4.194 ± 0.510 | 0.000 | 0.959 | 37 | 4.212 ± 0.457 | 0.004 | 0.823 |
| | AG | 167 | 4.189 ± 0.537 | | | 54 | 4.232 ± 0.565 | | |
| | GG | 75 | 4.172 ± 0.524 | | | 17 | 4.139 ± 0.595 | | |
| rs738409 | CC | 209 | 4.164 ± 0.536 | 0.013 | 0.089 | 61 | 4.155 ± 0.541 | 0.077 | 0.015 |
| | CG | 148 | 4.184 ± 0.519 | | | 44 | 4.229 ± 0.465 | | |
| | GG | 25 | 4.407 ± 0.395 | | | 3 | 5.052 ± 0.707 | | |
| rs1049728 | GG | 343 | 4.185 ± 0.520 | 0.001 | 0.763 | 93 | 4.220 ± 0.511 | 0.003 | 0.864 |
| | GC | 38 | 4.217 ± 0.560 | | | 14 | 4.139 ± 0.683 | | |
| | CC | 1 | 3.850 | | | 1 | 4.264 | | |

**Table I.** Association of *ICAM1* and *PNPLA3* polymorphisms with gene expression. Data accessed via HaploReg v4.1 (http://archive.broadinstitute.org/mammals/haploreg/haploreg.php).

| **Polymorphism** | **Chromosomal position (hg38)** | **Gene** | **Location** | **Tissue** | **Correlated gene** | ***P* value** | **Reference** |
|---|---|---|---|---|---|---|---|
| rs1799969 | Chr19: 10,284,116 | *ICAM1* | Exon 4 | Whole blood | *ICAM4* | $8.9 \times 10^{-11}$ | [3] |
| | | | | Whole blood | *ICAM3* | $3.9 \times 10^{-07}$ | [3] |
| | | | | Whole blood | *CDC37* | $5.8 \times 10^{-04}$ | [3] |
| rs5498 | Chr19: 10,285,007 | *ICAM1* | Exon 6 | Whole blood | *ICAM4* | $3.2 \times 10^{-37}$ | [3] |
| | | | | Blood | *ICAM4* | $1.4 \times 10^{-13}$ | [21] |
| | | | | Subcutaneous adipose tissue | *ICAM1* | $4.2 \times 10^{-10}$ | [22] |
| | | | | Transformed fibroblasts | *ICAM5* | $8.4 \times 10^{-10}$ | [1] |
| | | | | Whole blood | *ICAM4* | $1.5 \times 10^{-08}$ | [1] |
| | | | | Lymphoblastoid cell line | *ICAM4* | $1.0 \times 10^{-07}$ | [2] |
| | | | | Thyroid | *ICAM5* | $1.4 \times 10^{-07}$ | [1] |
| | | | | Omental adipose tissue | *ICAM1* | $2.1 \times 10^{-06}$ | [22] |
| | | | | Omental adipose tissue | *ICAM4* | $4.4 \times 10^{-06}$ | [22] |
| | | | | Omental adipose tissue | *ICAM5* | $3.2 \times 10^{-05}$ | [22] |
| rs738409 | Chr22: 43,928,847 | *PNPLA3* | Exon 3 | Whole blood | *SAMM50* | 0.0013 | [3] |

**Table J.** Analysis of *VCAM1* genotypes with log(soluble VCAM-1) levels stratified by obstructive sleep apnea status.

| SNP | Genotype | Obstructive sleep apnea | | | | No obstructive sleep apnea | | | |
|---|---|---|---|---|---|---|---|---|---|
| | | N | Mean (± SD) log(soluble ICAM1) level | $R^2$ | *P* value | N | Mean (± SD) log(soluble ICAM1) level | $R^2$ | *P* value |
| rs1582091 | GG | 92 | 6.851 ± 0.228 | 0.009 | 0.176 | 29 | 6.805 ± 0.198 | 0.012 | 0.539 |
| | GT | 206 | 6.796 ± 0.216 | | | 49 | 6.752 ± 0.199 | | |
| | TT | 84 | 6.800 ± 0.300 | | | 30 | 6.749 ± 0.282 | | |
| rs3176860 | AA | 129 | 6.840 ± 0.226 | 0.010 | 0.158 | 35 | 6.793 ± 0.180 | 0.008 | 0.669 |
| | AG | 193 | 6.788 ± 0.217 | | | 53 | 6.749 ± 0.224 | | |
| | GG | 60 | 6.817 ± 0.323 | | | 20 | 6.762 ± 0.292 | | |
| rs3176861 | CC | 228 | 6.819 ± 0.242 | 0.002 | 0.662 | 65 | 6.771 ± 0.219 | 0.005 | 0.759 |
| | CT | 131 | 6.796 ± 0.223 | | | 38 | 6.747 ± 0.245 | | |
| | TT | 22 | 6.793 ± 0.322 | | | 4 | 6.825 ± 0.133 | | |
| rs3176863 | GG | 269 | 6.812 ± 0.238 | 0.005 | 0.396 | 74 | 6.775 ± 0.209 | 0.077 | 0.015 |
| | GA | 104 | 6.813 ± 0.214 | | | 30 | 6.705 ± 0.227 | | |
| | AA | 8 | 6.695 ± 0.535 | | | 4 | 7.038± 0.309 | | |
| rs3176869 | AA | 284 | 6.808 ± 0.223 | 0.001 | 0.853 | 73 | 6.752 ± 0.227 | 0.018 | 0.386 |
| | AT | 93 | 6.816 ± 0.292 | | | 30 | 6.813 ± 0.226 | | |
| | TT | 3 | 6.742 ± 0.183 | | | 2 | 6.671 ± 0.085 | | |
| rs3176874 | AA | 285 | 6.795 ± 0.236 | 0.014 | 0.072 | 85 | 6.774 ± 0.231 | 0.008 | 0.650 |
| | AG | 87 | 6.847 ± 0.250 | | | 22 | 6.740 ± 0.200 | | |
| | GG | 9 | 6.927 ± 0.251 | | | 1 | 6.609 | | |
| rs3176877 | TT | 138 | 6.783 ± 0.246 | 0.015 | 0.060 | 44 | 6.772 ± 0.204 | 0.001 | 0.946 |
| | TA | 187 | 6.811 ± 0.238 | | | 52 | 6.757 ± 0.257 | | |
| | AA | 54 | 6.874 ± 0.229 | | | 11 | 6.757 ± 0.128 | | |
| rs3181088 | CC | 275 | 6.805 ± 0.245 | 0.007 | 0.247 | 72 | 6.748 ± 0.220 | 0.013 | 0.511 |
| | CT | 96 | 6.812 ± 0.210 | | | 34 | 6.796 ± 0.236 | | |
| | TT | 11 | 6.928 ± 0.336 | | | 2 | 6.852 ± 0.129 | | |
| rs3917009 | CC | 327 | 6.809 ± 0.239 | 0.001 | 0.880 | 93 | 6.767 ± 0.224 | 0.000 | 0.876 |
| | CT | 53 | 6.814 ± 0.249 | | | 15 | 6.757 ± 0.234 | | |
| | TT | 2 | 6.893 ± 0.296 | | | 0 | | | |
| rs6660837 | CC | 192 | 6.807 ± .0259 | 0.001 | 0.871 | 68 | 6.757 ± 0.240 | 0.004 | 0.806 |
| | AC | 154 | 6.816 ± 0.227 | | | 35 | 6.774 ± 0.206 | | |
| | AA | 33 | 6.794 ± 0.188 | | | 5 | 6.819 ± 0.118 | | |

**Table K.** Polymorphisms in this study previously associated with cardiovascular disease traits.

| Trait | Gene | Variant | Effect allele | *P* value | Odds ratio | Reference |
|---|---|---|---|---|---|---|
| Ischemic heart diseases | *RELA* | rs1049728 | C | 0.04 | 1.03 | [23] |
| Coronary artery disease | *ABO* | rs579459 | C | $2.7\times10^{-08}$ | 1.07 | [24] |
| Coronary artery disease | *ABO* | rs579459 | T | $1.1\times10^{-07}$ | 1.25 | [25] |
| Heart failure | *ABO* | rs579459 | T | $1.2\times10^{-06}$ | 0.90 | [23] |
| Heart attack/myocardial infarction | *ABO* | rs579459 | T | 0.0001 | 0.94 | [23] |
| Ischemic heart diseases | *ABO* | rs579459 | T | 0.0006 | 0.97 | [23] |
| Atherosclerosis | *ABO* | rs579459 | T | 0.001 | 0.86 | [23] |
| Heart/cardiac problem | *ABO* | rs579459 | T | 0.01 | 0.97 | [23] |
| Heart attack/myocardial infarction | *VCAM1* | rs6660837 | A | 0.04 | 1.03 | [23] |
| Heart/cardiac problem | *PNPLA3* | rs738409 | C | 0.003 | 1.03 | [23] |
| Ischemic heart diseases | *PNPLA3* | rs738409 | C | 0.02 | 1.02 | [23] |
| Heart failure | *ABO* | rs8176719 | T | $1.3\times10^{-05}$ | 0.92 | [23] |
| Heart attack/myocardial infarction | *ABO* | rs8176719 | T | 0.0001 | 0.95 | [23] |
| Atherosclerosis | *ABO* | rs8176719 | T | 0.002 | 0.88 | [23] |
| Ischemic heart diseases | *ABO* | rs8176719 | T | 0.006 | 0.98 | [23] |
| Heart/cardiac problem | *ABO* | rs8176719 | T | 0.01 | 0.98 | [23] |
| Coronary artery disease | *VCAM1* | rs3176863 | A | 0.03 | 1.10 | [25] |
| Postoperative myocardial infarction | *ICAM1* | rs5498 | G | 0.009 | 1.88 | [26] |

**Fig A.** Relationship between ABO blood groups and soluble E-selectin level in the current study as well as Blann *et al*.[27] and Qi *et al*.[7]

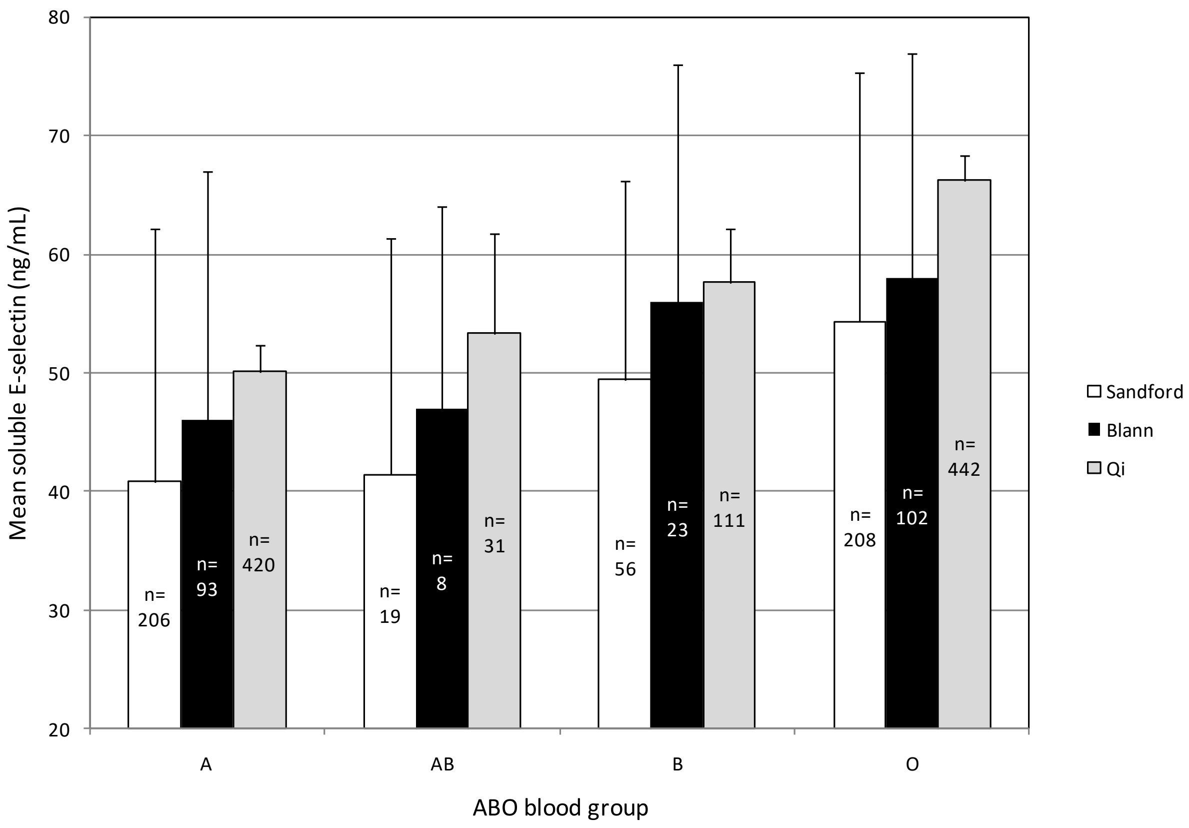

## References


1. GTEx Consortium. Human genomics. The Genotype-Tissue Expression (GTEx) pilot analysis: multitissue gene regulation in humans. Science. 2015;348: 648-660.
2. Lappalainen T, Sammeth M, Friedlander MR, t Hoen PA, Monlong J, Rivas MA, et al. Transcriptome and genome sequencing uncovers functional variation in humans. Nature. 2013;501: 506-511.
3. Westra HJ, Peters MJ, Esko T, Yaghootkar H, Schurmann C, Kettunen J, et al. Systematic identification of trans eQTLs as putative drivers of known disease associations. Nat Genet. 2013;45: 1238-1243.
4. Paré G, Ridker PM, Rose L, Barbalic M, Dupuis J, Dehghan A, et al. Genome-wide association analysis of soluble ICAM-1 concentration reveals novel associations at the *NFKBIK*, *PNPLA3*, *RELA*, and *SH2B3* loci. PLoS Genet. 2011;7: e1001374.
5. Paré G, Chasman DI, Kellogg M, Zee RY, Rifai N, Badola S, et al. Novel association of ABO histo-blood group antigen with soluble ICAM-1: results of a genome-wide association study of 6,578 women. PLoS Genet. 2008;4: e1000118.
6. Zhou L, He M, Mo Z, Wu C, Yang H, Yu D, et al. A genome wide association study identifies common variants associated with lipid levels in the Chinese population. PLoS One. 2013;8: e82420.
7. Qi L, Cornelis MC, Kraft P, Jensen M, van Dam RM, Sun Q, et al. Genetic variants in ABO blood group region, plasma soluble E-selectin levels and risk of type 2 diabetes. Hum Mol Genet. 2010;19: 1856-1862.
8. Li J, Gui L, Wu C, He Y, Zhou L, Guo H, et al. Genome-wide association study on serum alkaline phosphatase levels in a Chinese population. BMC genomics. 2013;14: 684.
9. Williams FM, Carter AM, Hysi PG, Surdulescu G, Hodgkiss D, Soranzo N, et al. Ischemic stroke is associated with the ABO locus: the EuroCLOT study. Annals of neurology. 2013;73: 16-31.
10. Shin SY, Fauman EB, Petersen AK, Krumsiek J, Santos R, Huang J, et al. An atlas of genetic influences on human blood metabolites. Nat Genet. 2014;46: 543-550.
11. Kim YJ, Go MJ, Hu C, Hong CB, Kim YK, Lee JY, et al. Large-scale genome-wide association studies in East Asians identify new genetic loci influencing metabolic traits. Nat Genet. 2011;43: 990-995.
12. Benyamin B, Esko T, Ried JS, Radhakrishnan A, Vermeulen SH, Traglia M, et al. Novel loci affecting iron homeostasis and their effects in individuals at risk for hemochromatosis. Nature communications. 2014;5: 4926.
13. Paterson AD, Lopes-Virella MF, Waggott D, Boright AP, Hosseini SM, Carter RE, et al. Genome-wide association identifies the ABO blood group as a major locus associated with serum levels of soluble E-selectin. Arterioscler Thromb Vasc Biol. 2009;29: 1958-1967.
14. Barbalic M, Dupuis J, Dehghan A, Bis JC, Hoogeveen RC, Schnabel RB, et al. Large-scale genomic studies reveal central role of ABO in sP-selectin and sICAM-1 levels. Hum Mol Genet. 2010;19: 1863-1872.
15. Chambers JC, Zhang W, Sehmi J, Li X, Wass MN, Van der Harst P, et al. Genome-wide association study identifies loci influencing concentrations of liver enzymes in plasma. Nat Genet. 2011;43: 1131-1138.
16. van der Harst P, Zhang W, Mateo Leach I, Rendon A, Verweij N, Sehmi J, et al. Seventy-five genetic loci influencing the human red blood cell. Nature. 2012;492: 369-375.
17. Rueedi R, Ledda M, Nicholls AW, Salek RM, Marques-Vidal P, Morya E, et al. Genome-wide association study of metabolic traits reveals novel gene-metabolite-disease links. PLoS Genet. 2014;10: e1004132.
18. Chung CM, Wang RY, Chen JW, Fann CS, Leu HB, Ho HY, et al. A genome-wide association study identifies new loci for ACE activity: potential implications for response to ACE inhibitor. The pharmacogenomics journal. 2010;10: 537-544.

19. Kamatani Y, Matsuda K, Okada Y, Kubo M, Hosono N, Daigo Y, et al. Genome-wide association study of hematological and biochemical traits in a Japanese population. Nat Genet. 2010;42: 210-215.
20. Teslovich TM, Musunuru K, Smith AV, Edmondson AC, Stylianou IM, Koseki M, et al. Biological, clinical and population relevance of 95 loci for blood lipids. Nature. 2010;466: 707-713.
21. Fehrmann RS, Jansen RC, Veldink JH, Westra HJ, Arends D, Bonder MJ, et al. Trans-eQTLs reveal that independent genetic variants associated with a complex phenotype converge on intermediate genes, with a major role for the HLA. PLoS Genet. 2011;7: e1002197.
22. Greenawalt DM, Dobrin R, Chudin E, Hatoum IJ, Suver C, Beaulaurier J, et al. A survey of the genetics of stomach, liver, and adipose gene expression from a morbidly obese cohort. Genome research. 2011;21: 1008-1016.
23. Canela-Xandri O, Rawlik K, Tenesa A. An atlas of genetic associations in UK Biobank. Nat Genet. 2018;50: 1593-1599.
24. CARDIoGRAMplusC4D Consortium, Deloukas P, Kanoni S, Willenborg C, Farrall M, Assimes TL, et al. Large-scale association analysis identifies new risk loci for coronary artery disease. Nat Genet. 2013;45: 25-33.
25. Schunkert H, Konig IR, Kathiresan S, Reilly MP, Assimes TL, Holm H, et al. Large-scale association analysis identifies 13 new susceptibility loci for coronary artery disease. Nat Genet. 2011;43: 333-338.
26. Podgoreanu MV, White WD, Morris RW, Mathew JP, Stafford-Smith M, Welsby IJ, et al. Inflammatory gene polymorphisms and risk of postoperative myocardial infarction after cardiac surgery. Circulation. 2006;114: I275-281.
27. Blann AD, Daly RJ, Amiral J. The influence of age, gender and ABO blood group on soluble endothelial cell markers and adhesion molecules. Br J Haematol. 1996;92: 498-500.
